# Macromolecular Crowding Directs Extracellular Matrix Organization and Mesenchymal Stem Cell Behavior

**DOI:** 10.1371/journal.pone.0037904

**Published:** 2012-05-23

**Authors:** Adam S. Zeiger, Felicia C. Loe, Ran Li, Michael Raghunath, Krystyn J. Van Vliet

**Affiliations:** 1 Department of Materials Science and Engineering, Massachusetts Institute of Technology, Cambridge, Massachusetts, United States of America; 2 Biosystems and Micromechanics Interdisciplinary Research Group (BioSyM), Singapore-MIT Alliance in Research and Technology (SMART), Singapore, Singapore; 3 Tissue Modulation Laboratory, Division of Bioengineering, Faculty of Engineering, National University of Singapore, Singapore, Singapore; 4 Graduate Programme in Science and Integrative Engineering (NGS), The National University of Singapore, Singapore, Singapore; 5 Department of Biological Engineering, Massachusetts Institute of Technology, Cambridge, Massachusetts, United States of America; 6 Department of Biochemistry, Yong Loo Ling School of Medicine, Singapore, Singapore; National Center for Scientific Research Demokritos, Greece

## Abstract

Microenvironments of biological cells are dominated *in vivo* by macromolecular crowding and resultant excluded volume effects. This feature is absent in dilute *in vitro* cell culture. Here, we induced macromolecular crowding *in vitro* by using synthetic macromolecular globules of nm-scale radius at physiological levels of fractional volume occupancy. We quantified the impact of induced crowding on the extracellular and intracellular protein organization of human mesenchymal stem cells (MSCs) via immunocytochemistry, atomic force microscopy (AFM), and AFM-enabled nanoindentation. Macromolecular crowding in extracellular culture media directly induced supramolecular assembly and alignment of extracellular matrix proteins deposited by cells, which in turn increased alignment of the intracellular actin cytoskeleton. The resulting cell-matrix reciprocity further affected adhesion, proliferation, and migration behavior of MSCs. Macromolecular crowding can thus aid the design of more physiologically relevant *in vitro* studies and devices for MSCs and other cells, by increasing the fidelity between materials synthesized by cells *in vivo* and *in vitro*.

## Introduction

Mesenchymal stromal or stem cells (MSCs) exhibit the capacity to self-renew and, through *in vitro* induction, to differentiate into multiple tissue cell lineages [1] including bone, cartilage, and fat. Such cells derived from human bone marrow can be isolated and expanded *in vitro*, and are therefore feasible for clinical applications such as tissue regeneration [1]. However, the potential consequences of current *in vitro* isolation approaches and expansion conditions for these cells [2], as well as the exact function of such MSCs *in vivo* after re-administration to the body [3], are not well understood. Indeed, the exquisite sensitivity of stem cells to changes in the extracellular environment [4] renders MSC identity and behavior challenging to characterize and manipulate *in vitro*.

For stem, progenitor, and differentiated tissue cell types, cell-material interactions are critical to the adhesion, proliferation, and migration of cells in both physiological and pathological states [3], though the mechanisms driving these interactions remain elusive [5], [6], [7]. Historically, the materials comprising the *in vitro* extracellular environment reflects a compromise between the critical *in vivo* physical and chemical properties that facilitate cell proliferation and the simplified conditions amenable to large-scale repetition and facile observation of cell response. This tension is particularly apparent in the solid materials with which cells interact: MSCs and other adherent cell types are routinely isolated and expanded in number on optically transparent glass or polystyrene surfaces, which are topographically flat and also orders of magnitude stiffer than extracellular matrices *in vivo*. However, isolation, expansion, and phenotypic differentiation of MSCs in particular have been correlated with mechanical cues (e.g., stiffness [8] or topography of an underlying substratum [9] or three-dimensional scaffold [10], or interstitial flow [11]), as well as more well-studied biochemical cues (e.g., soluble growth factors and tethered ligand density [7]). Thus, most recent efforts to study and engineer the function of MSCs and other animal cells *in vitro* have focused on the solid materials to which these cells adhere. In contrast, the macromolecular nature of the fluid component of *in vitro* microenvironments has been much less scrutinized. We show here that the simplifications adopted in the vast majority of *in vitro* studies result in interesting consequences for the organization of and reciprocity between cells and their microenvironments.

Indeed, it has been largely overlooked that the *in vivo* soluble environment exhibits a much higher overall concentration of several macromolecular species than the *in vitro* counterpart [12]. Again, for reasons of practical convenience and well-controlled aqueous environments, the fluid component of *in vitro* cell studies comprises a so-called essential medium defined by concentrations of specific ions, amino acids, and growth-factor proteins; this medium is supplemented by an ill-defined but vital mixture of animal serum proteins [13]. The serum admixture, or protein additions that replace specific serum components, contribute the sole macromolecular element of *in vitro* culture (typically 1–20 vol%). However, the high dilution of serum proteins in the final culture medium results in much lower concentration (1–10 g/L or lower [12]) than in interstitial fluids (30–70 g/L [14], [15]), blood plasma (80 g/L [16]), or the cell interior (200–350 g/L [12]). The concept of macromolecular crowding effects is well described in material physics and is attributed to an excluded volume effect by which even low concentrations of small macromolecules can induce entropic segregation of other molecules [17]. Generally, the addition of as little as 1 vol% macromolecules to the *in vitro* environment sufficiently crowds the functional proteins of interest to accelerate biochemical reactions and assembly – including enzymatic and polymerization reaction rates, binding and folding kinetics, and gelation and protein fibril formation [18], [19] – to approach *in vivo* levels. As a result, many theoretical and experimental studies of macromolecular crowding in protein solutions have posited that consequences may exist for cell-material interactions. However, to our knowledge, the effects of macromolecular crowding on the cell and matrix materials have not yet been demonstrated directly. To better understand the role of extracellular macromolecules, we investigated the impact of macromolecular crowding on organization of the intra- and extracellular protein networks, as well as on specific behaviors of non-differentiating MSCs. We posit a reciprocity between the cells and the matrix material, whereby crowding directly affects matrix architecture and thus indirectly alters intracellular organization, matrix protein production, and cell behavior.

## Results and Discussion

We increased the degree of extracellular crowding by adding Ficoll®, a specific macromolecular crowding agent, directly to basal cell-culture media. Ficoll is a non-interacting, neutral polymer comprising a copolymerization of sucrose and epichlorhydrine; this epoxide-linked polysaccharide exhibits a mean hydrodynamic radius of ∼4 nm (Fc70) or ∼8 nm (Fc400) [20], and is commonly utilized as a vitrification agent and in gradient centrifugation of cells. It is non-cytotoxic and does not significantly impact solution viscosity at relevant concentrations [21]. Further, unlike other macromolecules that have been considered crowding agents (e.g., dextran sulfate), Ficoll is net charge-neutral and thus does not conflate the excluded volume effects of a crowding agent [22] with the charge-based aggregation of proteins. Lareu et al. have demonstrated previously that addition of this macromolecule to the culture media of fibroblasts accelerated the enzymatic conversion of pro-collagen to collagen [23], [24], a crucial prerequisite for collagen matrix formation. However, the impact of this change on extracellular matrix (ECM) structure or on cell structure or function was not considered. In the present study, adult human MSCs were seeded at low density (∼7500 cells/mL or ∼2100 cells/cm^2^) in complete basal culture media; media was replaced 24 h after seeding, with or without addition of 62.5 mg/mL Ficoll mixture (see Methods) that is referred to hereafter as MMCs (macromolecular crowders). This crowding concentration represents the same order of magnitude of the total protein concentration found in blood plasma (80 mg/mL) [25]. More relevant to considerations of excluded volume effects, the corresponding fractional volume occupancy, defined as the fraction of total volume occupied by crowder macromolecules, is 17 vol%; this degree of crowding is representative of the perfused bone marrow compartment [20], [26].

**Figure 1:**
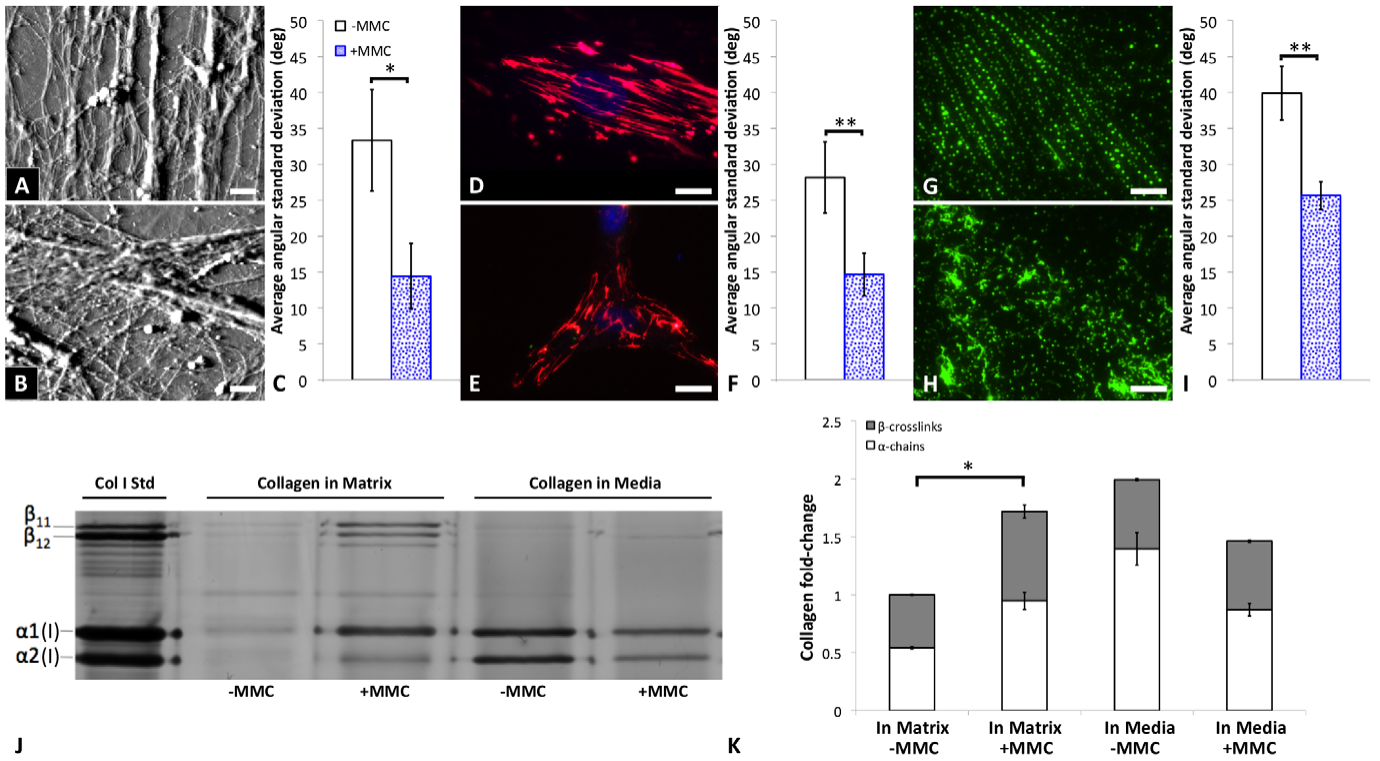
Macromolecular crowding induces alignment of extracellular matrix fibers and an increase in deposition of collagen type-I in human bone marrow-derived mesenchymal stromal or stem cells (MSCs). (**A**) Atomic force microscopy (AFM) contact mode deflection images of extracellular matrix deposited by MSCs left behind after detergent removal of cells in media containing macromolecular crowders (+MMC media) and (**B**) −MMC media after 7 days. Scale bars = 2 mm. (**C**) Average angular standard deviation for AFM imaging of extracellular matrix in **A** and **B** (N = 5 +MMC, N = 5 −MMC, p = 0.1363). (**D**) Immunostaining of extracellular fibronectin (red) and cell nucleus (blue, DAPI) for +MMC and (**E**) −MMC in MSCs after 3 days of culture. Scale bars = 30 µm. (**F**) Average angular standard deviation for fibronectin in **D** and **E** (N = 15 +MMC, N = 11 −MMC, p = 0.0013). (**G**) Immunostaining of extracellular collagen IV (green) and cell nucleus (blue, DAPI) +MMC, (**H**) and −MMC, in hMSCs after 3 days of culture. Scale bars = 15 mm (**I**) Average angular standard deviation for collagen IV in **G** and **H** (N = 12 +MMC, N = 8 −MMC, p = 0.0253). Lower values of average angular standard deviation indicate a higher degree of alignment of fibers comprising the extracellular matrix. (**J**) Western blot of collagen type-I secreted by human bone marrow-derived mesenchymal stromal or stem cells after 48 hrs in culture medium ± MMC demonstrates a significant increase in the cell deposited collagen into the matrix +MMC. (**K**) Normalized densitometry of collagen type-I Western blot demonstrates a shift in collagen distribution from media to matrix, indicative of the enhanced deposition of collagen in the presence of MMCs. Significant increases in crosslinked collagen α-chains (β-bands) is observed in the cell deposited matrix +MMC, as compared to −MMC conditions (p<0.0001). Values are reported as mean ± standard error of measurement. * indicates p = 0.1363; and ** indicates statistical significance (p<0.05).

**Figure 1** shows the organization of the extracellular matrix when crowding is induced within the culture media. First, to characterize the matrix independently of the cells, MSCs were removed at day 7 via deoxycholate lysis buffer, retaining the ECM structure to enable imaging via atomic force microscopy (AFM, fully hydrated in aqueous solution). Organization of the extracellular matrix networks was quantified as the average angular standard deviation <α_sd_> among filaments in such images. This approach captures the distribution width of fibril alignment in networks [27], where lower <α_sd_> indicates increased degree of alignment, independent of relative fibril orientation (see Methods and Figure S1). As demonstrated by **Figures 1A–C**, the ECM deposited by cells cultured in crowded media conditions (+MMC) exhibited an increased degree of alignment of ECM fibers (p = 0.1363). However, ECM deposited by MSCs cultured in standard media conditions (−MMC) also resulted in poorly adsorbed ECM after lysing, ostensibly due to the proposed enhancement of surface attraction under crowded conditions [28]. Second, immunochemical staining of the intact ECM showed significantly increased alignment of both fibronectin (**Figures 1D–F**, p<0.001) and collagen IV (**Figures 1G–I**, p<0.03) under induced macromolecular crowding. Here, the intact ECM was primarily co-located with and surrounding cells; image locations for alignment quantification were thus selected randomly via 4′,6-diamidino-2-phenylindole (DAPI) nuclear DNA staining. Similar differences in matrix alignment were observed regardless of whether +MMC media was introduced 24 h after cell seeding (as in Figure 1) or at the initial seeding (data not shown). In summary, significant increases in ECM alignment are observed in crowded media conditions.

Enhanced physisorption of macromolecules including matrix proteins from solution to surfaces is a general hallmark of macromolecular crowding [22]. To determine whether MSCs produced and deposited more matrix proteins in the presence of MMCs, we compared amounts of collagen deposited with amounts of collagen secreted into the media. **Figure 1J** shows via Western blot that more collagen type-I was deposited by MSCs under +MMC conditions within 48 h, in agreement with earlier findings [24]. Further, normalized densitometry (**Figure 1K**) demonstrates a shift in collagen distribution from the media to the matrix under +MMC conditions. Specifically, the relative fraction of crosslinked collagen (β-bands) increases with respect to the uncrosslinked collagen (α-bands) in the ECM deposited by MSCs under crowded conditions. This is consistent with previous findings that crowding accelerates procollagen conversion and lysyl oxidase-mediated crosslinking [29]. Similar results were also observed for fibronectin (Figure S2), and have been reported for other crowding agents and cell types [30]. These data show that the amount and crosslinked fraction of a specific ECM protein are increased in crowded media conditions.

**Figure 2:**
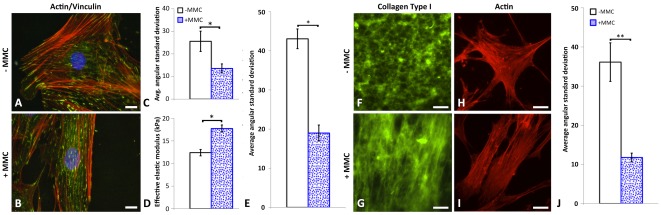
Macromolecular crowding directly alters organization of deposited extracellular matrix proteins and thus alters the orientation of the actin cytoskeleton. (**A**) Immunostaining of intracellular F-actin (red), intracellular vinculin (green) as a focal adhesion protein involved in the linking of integrin to actin cytoskeleton, and nucleus (blue, DAPI) of human bone marrow-derived mesenchymal stromal or stem cells (MSCs) after 3 days of cell culture in media containing macromolecular crowders (+MMC media) and (**B**) −MMC media. Scale bars = 30 µm. (**C**) Quantification of average angular standard deviation for F-actin (N = 10 +MMC, N = 10 −MMC, p = 0.0223) where lower values indicate a higher degree of alignment. (**D**) Effective Young's elastic modulus in kPa measured by atomic force microscopy enabled nanoindentation of MSCs ± MMC suggests a stiffening of the cortical cytoskeleton +MMC. (**E**) Average angular standard deviation of FITC-conjugated rat tail type-I collagen network deposited on plasma treated glass coverslips, (**F**) in media absent of macromolecular crowders (−MMC) and, (**G**) +MMC. Scale bars = 25 µm. (**H**) Immunostaining of F-actin (red) after 3 days for human bone marrow-derived mesenchymal stromal or stem cells cultured in basal −MMC media seeded onto type-I collagen networks formed under −MMC, or (**I**) +MMC conditions (N = 13 +MMC, N = 13 −MMC, p = 0.0001). Scale bars = 25 µm. (**J**) Average angular standard deviation of actin fibers for **H** and **I**. Values are reported as mean ± standard error of measurement. * indicates statistical significance (p<0.05). ** indicates statistical significance (p<0.001).

Figure 1 illustrates the effect of MMCs on organization of extracellular protein networks. To investigate the influence of MMCs on the organization of intracellular networks, we quantified the angular distribution of the cytoskeletal F-actin via immunocytochemical staining (**Figures 2A–B**). As in the above characterization of the ECM, image locations were selected randomly via DAPI nuclear DNA staining, to avoid biased analysis of cells exhibiting any particular morphology or orientation. **Figure 2C** shows that <α_sd_> of the actin cytoskeleton decreased significantly for MSCs under +MMC conditions (p<0.02). This increased alignment of the cytoskeleton was thus observed concomitantly with increased alignment of the extracellular matrix. Further, **Figure 2D** indicates that the stiffness of the MSC cortical cytoskeleton, as quantified via AFM-enabled nanoindentation of these cells at 37°C, indented to a maximum depth of 20 nm, also increased significantly with macromolecular crowding (p<0.0001). This local stiffening is consistent with the increased alignment of the actin cytoskeleton in MSCs and other adherent cell types [2].

Although Ficoll is considered a neutral, non-interacting macromolecule, we explored whether it would reach the cell interior via endocytosis, thereby inducing crowding within the cell and altering cytoskeletal alignment independently of matrix alignment. We observed only minor uptake of both molecular weights of Ficoll by MSCs over 24 h, and confirmed via calcein labeling that these punctate regions did not release MMCs into the cytoplasm. Instead, MMCs remained confined within vesicles of mm-scale diameter (Figure S3). Further, even in dilute media conditions, the cell interior is already a highly crowded environment of total macromolecular concentration >200 mg/mL [31]. It is therefore unlikely that minor uptake of this crowder within vesicles significantly increased the degree of intracellular crowding.

It has been shown previously that dilute polymer solutions can undergo a transition with increasing concentrations of polymer, from disordered crosslinked networks to entropically demixed or more ordered phases similar to the nematic phase of liquid crystals [32]. To consider whether *in vitro* crowding of ECM proteins could directly produce aligned networks in the absence of adherent cells, we generated fluorescently labeled collagen matrices from pure collagen solutions, deposited on plasma-treated glass coverslips in the absence or presence of this same concentration and composition of the MMCs. We observed that addition of the MMCs to 0.5 mg/mL collagen resulted in a significant change in network orientation (p<0.0001), with increased collagen alignment and a correspondingly decreased <α_sd_> (**Figures 2E–G**). When MSCs were seeded onto such paired unlabeled collagen matrices in the absence of MMCs in the culture media – i.e., seeded in uncrowded media onto a collagen matrix assembled under crowded conditions – cells exhibited a significantly increased actin cytoskeleton alignment (**Figures 2H–J**, p<0.0001) that was comparable to that exhibited by cells exposed only to crowded media (Figures 2A–B). These findings are consistent with the concept that macromolecular crowding promotes alignment of the extracellular matrix material at relatively low matrix protein concentrations, which in turn directs the organization of cell-matrix adhesion and cytoskeletal alignment. Further, we note that although other macromolecular crowding agents can also alter the organization of these collagen networks at similar fractional volume occupancy (Figure S4), these changes are typically conflated by additional factors such as charge-associated or hydrogen bonding-associated interactions with proteins [33].

**Figure 3:**
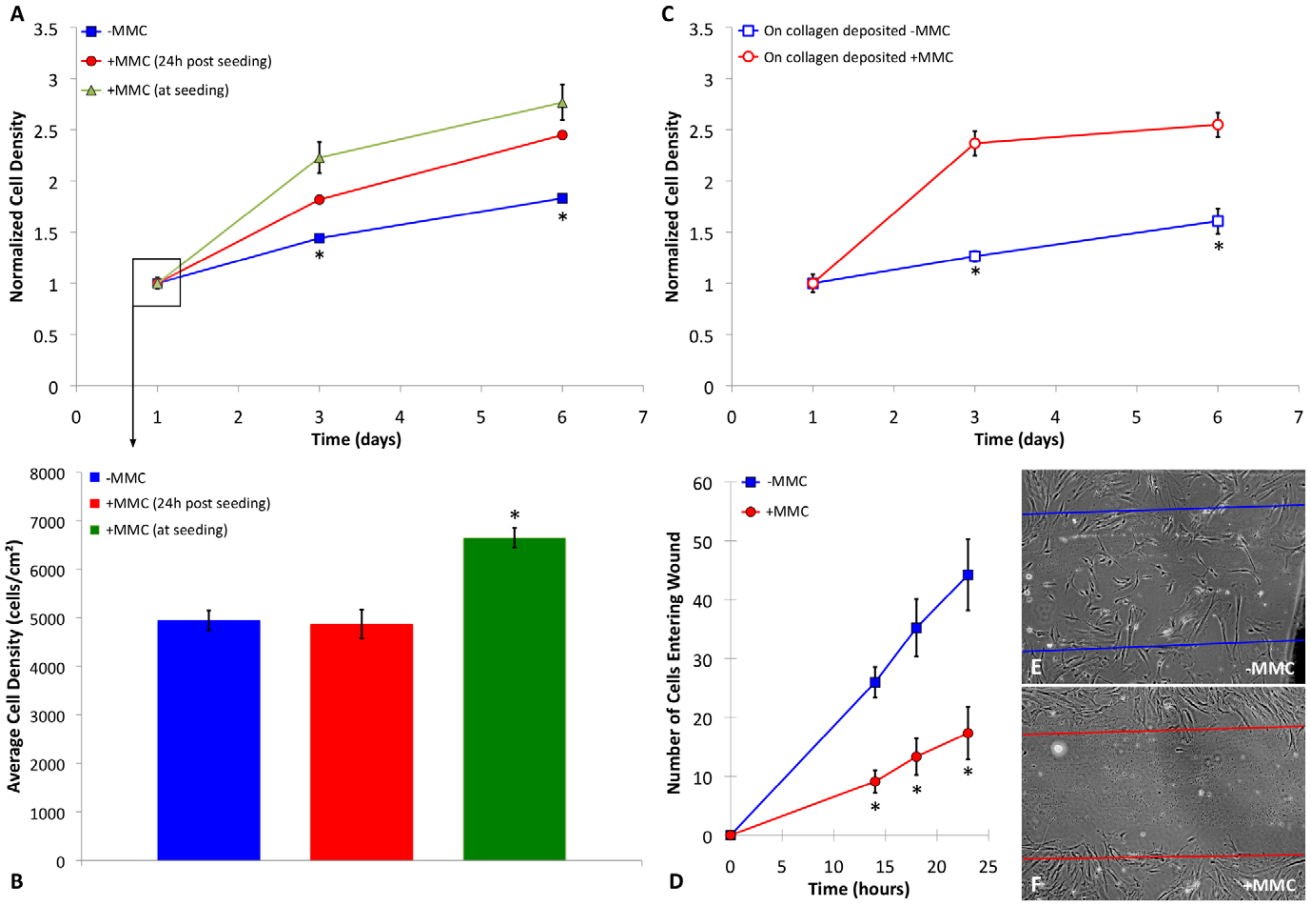
Consequences of altered extracellular matrix organization include enhanced proliferation, altered adhesion, and decreased migration. (**A**) Human bone marrow-derived mesenchymal stromal or stem cells (MSCs) cultured in media containing macromolecular crowders (+MMC media, filled red circle) demonstrate statistically significant increase in proliferation versus cells in −MMC media (filled blue square). Furthermore, cells seeded directly in +MMC media (filled green triangle) also demonstrate a similar increase in proliferation. (**B**) Cells seeded directly in +MMC media (green) demonstrate enhanced adhesion after 24 hours as compared to cells seeded without crowder (−MMC, blue +MMC (24 h post seeding), red). (**C**) MSCs cultured in basal media on collagen type-I deposited +MMC (open red circle) also exhibit similarly altered proliferation behavior compared to MSCs on collagen deposited −MMC (open blue square). (**D**) Migration of cells, as determined by a wound healing assay, is significantly reduced +MMC (open red circle) (**E**) as compared to −MMC (open blue square) (**F**) (N = 9). Values are reported as mean ± standard error of measurement. * indicates statistical significance (p<0.001).

Finally, to consider whether induction of crowding and the resulting increase in ECM alignment would affect cell behavior as well as cytoskeletal organization, we quantified MSC adhesion, proliferation, and migration in these *in vitro* environments. Cell adhesion and proliferation were quantified by seeding MSCs onto tissue culture-treated polystyrene at low density (∼2100 cells/cm^2^), with macromolecular crowders added during, or 24 hours after, initial seeding; whole-well cell counts were obtained via DAPI nuclear staining at 1, 3, and 7 days (see Methods). **Figure 3A** shows that MSC proliferation increased significantly (p<0.0001) by day 3 when crowding was induced 24 h post-seeding, and even more so when crowding was induced at initial cell seeding. This increased proliferation rate cannot be attributed to crowder-induced acceleration of DNA synthesis by cells, in that EdU assays of cells entering S-phase within 24 h was not statistically different in the presence of MMCs (data not shown). However, **Figure 3B** shows that MMCs also promoted initial adhesion of cells, with a significant increase in the number of cells adhered when seeded directly into crowded media. This finding is consistent with the increased production of matrix proteins by cells under crowded conditions and enhanced alignment of such proteins upon deposition under crowded conditions (Figure 1), as well as enhanced macromolecular adsorption to surfaces under crowded conditions [22]. Thus, enhanced proliferation when crowder was introduced at initial seeding, as compared to 24 hr later (Figure 3A), can be attributed to both the longer duration of crowding-facilitated matrix deposition and alignment at day 3, as well as the increased number of adhered cells at day 0. (It is established that MSC proliferation rates depend on initial cell seeding density [34].) This interpretation is further supported by **Figure 3C**, which shows that MSCs exhibited significantly increased proliferation (p<0.0001) when seeded onto matrices comprising collagen that was deposited with or without macromolecular crowders (as in Figure 2, but with unlabeled collagen). Here, no crowders were added to the cell culture media, and there was no significant difference in initial adhesion of MSCs to such matrices (p>0.4). **Figures 3D–F** shows that MSCs also exhibited significantly decreased migration efficiency (p<0.0001) in a classical *in vitro* wound healing assay [35] in which both the cell and matrix layers were mechanically removed from the surface, over 24 h in +MMC media. The reduced capacity of MSCs to migrate into a disrupted cell-matrix region is consistent with increased adhesion between the cells and the ECM that these cells deposited over ∼7 days *in vitro* prior to the wound (see Methods). This finding is further supported by oriented focal adhesions observed in Figures 2A and 2B, as well as a significant increase in effective Young's elastic modulus of the cortical actin cytoskeleton under macromolecularly crowded conditions, as demonstrated in Figure 2D. This finding is similar to the generally observed reduction in cell migration speed at sufficiently high concentration of adsorbed matrix proteins *in vitro* [36]. The present study was intentionally constrained to non-differentiating conditions of MSCs (see Figure S5), as differentiation is concomitant with complex matrix remodeling processes. However, as will be outlined separately [37], we have observed that the consequences of macromolecular crowding also extend to the *in vitro* differentiation potential of these cells.

Our understanding of stem cell characteristics and function, and therefore our progress in designing relevant *in vivo* applications, is derived primarily from *in vitro* cell culture models. Indeed, this intended correlation between *in vitro* material microenvironments and *in vivo* niches extends to nearly all cell types and cell responses quantified in laboratory settings. However, one important difference that we consider here is the orders-of-magnitude lower concentration of macromolecules *in vitro* [12], even when other microenvironment characteristics such as matrix stiffness, fluid flow rates, and specific protein concentrations are increasingly engineered to approach *in vivo* conditions. Macromolecular thermodynamics and kinetics in solution are significantly affected by excluded volume effects [38], [39], [40], [41], [42], and the cell exterior and interior are naturally crowded *in vivo* and subject to such excluded volume effects [43], [44], [45]. Thus, Ellis et al. have long argued that macromolecular crowding should be maintained explicitly when studying biological systems under physiologically relevant conditions [12], [46]. Indeed, it is well established that crowding via addition of more serum proteins is not a viable option, commonly resulting in cell overproliferation and other dysfunctions associated with superphysiological concentrations of growth factors and other proteins [47]. Therefore, synthetic polymeric crowding agents such as the MMCs considered here are desirable to minimize unintended biochemical interactions while inducing a level of macromolecular crowding commensurate with *in vivo* levels [48].

By inducing physiological levels of macromolecular crowding in the culture media of adult human bone marrow-derived MSCs, we identified clear correlations between the increased alignment and deposition of the extracellular matrix fibrillar networks (Figure 1), and the intracellular actin cytoskeleton (Figures 2A–D). These correlations in the presence of macromolecular crowding could be rationalized either by the cell guiding organization of the matrix material, or by the matrix guiding organization of the cytoskeleton. In other words, did MMCs primarily induce outside-in signaling from the material to the cell, or inside-out signaling from the cell to the matrix material [49]? We found that induction of crowding during deposition of collagen resulted in enhanced matrix alignment, even in the absence of cells (Figures 2E–G); and that cells that were subsequently deposited on such aligned collagen exhibited increased actin cytoskeleton alignment, even in the absence of MMCs in the media (Figures 2H–J). These findings are consistent with the concept that physiological levels of macromolecular crowding enhance matrix deposition and alignment, which in turn guides cytoskeletal actin alignment. This reciprocity is then manifested in associated increases in the cells' effective stiffness and matrix protein secretion, and potentially even the forces generated by the cell against the matrix material that may further promote ECM alignment.

**Figure 4:**
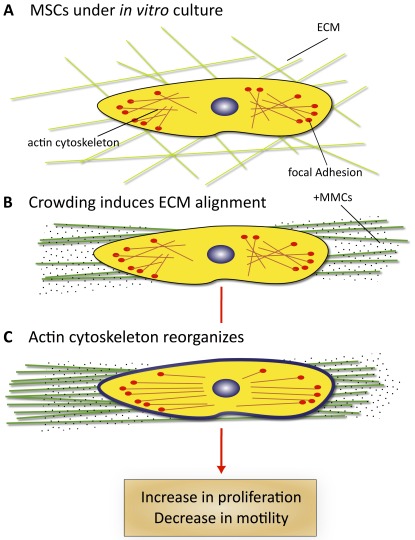
Schematic of cell and matrix reciprocity under macromolecular crowding. (**A**) Adherent mesenchymal stromal cell (MSC) under typical *in vitro* culture conditions. (**B**) Upon the addition of macromolecular crowders, excluded volume effects promote bundling and alignment of extracellular matrix (ECM) fibers. (**C**) In response to the aligned ECM, the intracellular actin cytoskeleton reorganizes to align with the ECM. In addition, the cell secretes more matrix proteins, which are deposited to and further enhance the alignment of the ECM. We find that these reciprocal changes in the matrix material and intracellular cytoskeletal organization correlate with an increase in proliferation and a decrease in motility of MSCs.

**Figure 4** summarizes this cell-matrix reciprocity under conditions of extracellular macromolecular crowding. Such chemomechanical coupling at the cell-material interface directly affects adhesion, morphology, and motility of adult human MSCs in the basal or undifferentiated state (Figure 3), and future studies can now consider whether *in vitro* differentiation potential into mesenchymal tissue lineage cells is also impacted. Note that this perspective does not obviate intracellular molecular signaling cascades as mediators of this cell-matrix reciprocity, but rather shows that physical interactions among extracellular macromolecules can significantly and independently modulate the local material environment of the cell [20], [26]. In fact, it is quite plausible that this increased matrix deposition and alignment are also highly correlated with local mechanical stiffness, densities of key tethered proteins, and other cues that have demonstrated correlations with cell morphology or function via increasingly detailed signal-response pathways [50]. However, these results demonstrate directly that induction of physiological crowding levels affects not only the long-noted increase in reaction rates among macromolecules in solution, but also the architecture and functionality of the cell-matrix environment. The induction of macromolecular crowding for *in vitro* systems enables one of the several critical components required to simulate *in vivo* niches with high fidelity, particularly via the extracellular material that is ultimately generated and maintained by the cells. Thus, increased consideration of such excluded volume effects in the laboratory can close the performance gap between engineered environments and biological niches, for applications ranging from basic cell science to stem cell delivery to tissue-based cancer therapies [26], [51], [52].

## Materials and Methods

### Cell Culture

Human bone-marrow derived mesenchymal stromal or “stem” cells (MSCs; ReachBio Seattle, WA) were expanded until passage 4–6, and plated onto 50 mm-diameter glass bottom Petri dishes (MatTek P50G-0-30-F Ashland, MA) or 60-mm diameter tissue culture polystyrene (Falcon 35-1006 Franklin Lakes, NJ) at low density (∼7500 cells/mL) to maintain subconfluent culture conditions. Cells were cultured in typical basal MSC culture media, which we refer to as “uncrowded media” (−MMC), comprising complete MesenCult medium (MesenCult basal medium, catalog #05401, plus with 20% MesenCult serum-containing Supplemental, catalog #05402; StemCell Technologies, Vancouver, BC) and 2 µM L-glutamine, 100 units/mL penicillin and 100 µL/mL streptomycin (Invitrogen, 15140-163, Carlsbad, CA). For “crowded media” (+MMC) an additional 62.5 mg/mL of crowding agents comprising Ficoll, a synthetic co-polymer of the polysaccharide sucrose and epichlorohydrin, of 37.5 mg/mL of 70 kDa and 25 mg/mL of 400 kDa (GE Healthcare Uppsala, Sweden) was added to the −MMC media. Media was exchanged 24 hrs after initial seeding with −MMC or +MMC media. Cells were maintained in 5% CO_2_ at 37°C. Media was exchanged after every 7 days for subculture and 3 days for orientation and proliferation assays.

Ficoll was chosen as the crowding agent due to its neutral charge and relatively small hydrodynamic radius. The hydrodynamic radius of Ficoll has been measured as 4 and 8 nm, for 70 kDa and 400 kDa molecular weights, respectively. Unlike other macromolecules that have been considered as crowding agents (e.g., dextran sulfate), Ficoll is net charge-neutral and thus does not conflate the exclude volume effects of a crowding agent [22]. Other crowders such as polyvinyl pyrrolidone (PVP, Sigma 856568) were investigated, but these crowders proved cytotoxic at relevant concentrations (data not shown).

### Matrices Assembled in Absence of Cells

For crowder-aligned collagen matrices that were used subsequently for cell seeding, 1 mL of 0.1 mg/mL rat tail collagen Type-I (BD Biosciences, cat. #354236) in Tris-HCl buffer at pH 7.4±62.5 mg/mL MMC was deposited onto 50 mm-diameter glass bottom Petri dishes (MatTek P50G-0-30-F Ashland, MA) and incubated at 37°C for 4 h. Matrices were rinsed three times with 137 mM NaCl phosphate buffered saline (PBS) before cell seeding.

For crowder-aligned fluorescently labeled collagen matrices that were used subsequently to quantify alignment of cell-free matrices, glass microscope slides (VWR, 16004) were ozone treated with a laboratory corona treater (Electro-technic Products, Inc. Chicago, IL) for 10 sec to increase hydrophilicity and enhance molecular adsorption. One mL of FITC-conjugated Type I Collagen (AnaSpec, 85111) was deposited on the plasma treated glass microscope slides (VWR, 16004) at 0.01 mg/mL or 0.5 mg/mL in 0.02 M acetic acid ±62.5 mg/mL of the MMC solution. Slides were then incubated at 37°C for 4 h.

Other crowders such as 50 mg/mL of polyvinyl pyrrolidone (PVP, Sigma 856568-5G), 50 mg/mL of bovine serum albumin (BSA, Sigma A7906-50G) and 50 mg/mL of polyethylene glycol (PEG 10000, Sigma 92897) in 0.5 mg/mL FITC-conjugated Type I collagen in 0.02 M acetic acid, were also explored.

### Western Blot

Cell layers were extracted with a buffer containing 150 mM NaCl, 50 mM Tris (pH 7.5), 5 mM EDTA (pH 8.0), 1% Triton-X100, and Protease Inhibitor Cocktail tablets (Roche Diagnostics Asia Pacific, Singapore). Twenty uL of protein extract for each sample were mixed with 1× Laemmli buffer and 2 mL of b-mercaptoethanol and subjected to small format SDS-PAGE. Proteins were then electroblotted onto a nitrocellulose membrane (Bio-Rad) with the Mini Trans-Blot transfer cell (Bio-Rad) according to the manufacturer's protocol. Membranes were blocked with 5% nonfatmilk (Bio-Rad) in TBST pH 8 (20 mMTris-base-150 mMNaCl-0.05%Tween-20) for 1 h at RT. Subsequently, the primary antibody (rabbit anti-human collagen I; abcam) at a 1∶500 dilution with 1% nonfat milk in TBST was incubated for 1 h at RT. Bound primary antibody was detected with goat anti-rabbit HRP (Pierce Biotechnology, Rockford, IL) diluted 1∶1000 in 1% nonfat milk in TBST for 1 h at RT. The membrane was then incubated with Super Signal West Dura substrate (Pierce Biotechnology) and chemiluminescence was captured via an LAS-1000 Luminescent Image Analyzer (Fuji Photo Film, Tokyo, Japan).

### Pepsin Digest

Extracellular media and cell-deposited extracellular matrix were analyzed by SDS-PAGE under non-reducing conditions (Mini-Protean 3; Bio-Rad Laboratories, Singapore). Protein bands were stained with SilverQuest (Invitrogen) according to the manufacturer's protocol. Densitometric analysis of wet gels was performed on the GS-800 Calibrated Densitometer (Bio-Rad) with Quantity One v4.5.2 image analysis software (Bio-Rad). Collagen bands were quantified by defining each band as a rectangle with local background subtraction.

### Immunocytochemistry

To assay orientation of fibronectin, collagen, and actin, cells were fixed using 4% paraformaldehyde (AlfaAesar 43368 Ward Hill, MA) in PBS for 15 min at room temperature after 24 h, 3 days, and 7 days of treatment. Following fixation, cells were washed briefly with PBS containing 0.05% Tween-20 (Teknova P1176 Hollister, CA) and permeabilized for 3 min at room temperature with 0.1% Triton X-100 (Fluka 93443, Switzerland). To minimize non-specific binding, cells were treated with 3% bovine serum albumin (Sigma, A7906) (BSA) in PBS for 30 min before staining. Cells were incubated at room temperature with relevant primary antibodies in 3% BSA for mouse anti-human fibronectin (Abcam26245, 1∶100) and rabbit anti-human collagen IV (Abcam6586, 1∶100), or monoclonal anti-vinculin (Sigma V4505, 1∶200). Cells were then incubated with secondary antibodies AlexaFluor 488 chicken anti-rabbit (Molecular Probes, A21441, 1∶400), AlexaFluor 594 goat anti-mouse (Molecular Probes, A11020, 1∶400), and goat anti-mouse IgG (Abcam6785, 1∶400) respectively. Cells stained for collagen IV or vinculin were also double labeled with Alexa Fluor 555 Phalloidin (Molecular Probes, A34055, 1∶1000) for 60 min. Cells were rinsed 3 times (10 min each) with PBS and imaged by fluorescence microscopy (IX-81, Olympus America, Inc.) and captured using Slidebook 4.0 (Intelligent Imaging Innovations, Inc. Denver, CO). Cell nuclei were also counterstained with 4′,6-diamidino-2-phenylindole (DAPI) (Millipore 90229, 1∶2000).

### Intracellular Uptake of Crowding Agents

To determine the nature of Ficoll uptake, cells were cultured with 1 mg/mL of TRITC- and FITC-labelled Ficoll (TdB Consultancy, Uppsala, Sweden) of molecular weights 70 kDa and 400 kDa, respectively, in −MMC media for 24 hours. Calcein (Sigma,C0875) was also added to the media at 0.24 µM, as previously described [53], to investigate the nature of vesicle release of Ficoll after endocytosis. After 24 h, cells were fixed and permeabilized, as described above, before imaging.

### Preparation of Lysed ECM for AFM Imaging

To generate ECM for AFM, MSCs were seeded at 3.5×10^4^ cells/well in 24-well plates (Celstar) and maintained in basal media ± MMC for 7 days prior to lysis. For lysis, monolayers were first washed with PBS twice then treated with 0.5% DOC (Prodotti Chimici E Alimentari, S.P.A. 2003030085) supplemented with 0.5× Complete Protease Inhibitor (Roche Diagnostics Asia Pacific, 11836145001) in water for 15 min at room temperature four times, followed by 0.5% DOC in PBS for 30 min at room temperature on a nutating platform. Monolayers were then incubated with DNAse (Sigma) for 1 hr at 37°C, then washed with PBS three times.

### Quantification of Protein Network Alignment

To quantify the orientation of fibers (fibronectin, collagen, or actin) in ±MMC conditions, images were analyzed via a customized MATLAB script which quantified the orientation vector magnitudes for each fiber, as described elsewhere [54]. The angular standard deviation, *SD*, is calculated by equation (1):
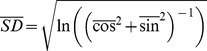
(1)where,
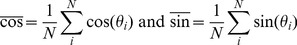
(2)where *θ_i_* is the angle of the *i* ^th^ fiber, and the bar superscript indicates the mean quantity [27]. The average angular standard deviation was reported for each fiber of interest, where a lower angular standard deviation indicates a higher order of alignment. All values of average angular standard deviation were reported as mean ± standard error of measurement.

### Cell Cycle Analysis

To determine if MMCs induced entrance into S-phase of the cell cycle, an EdU assay was conducted (Click-iT® EdU Alexa Fluor 488 Imaging Kit, C10337, Molecular Probes). Briefly, cells were labelled with 5-ethynyl- 2′-deoxyuridine (EdU) and incubated for 4 or 24 hours with −MMC or +MMC media at 37°C and 5% CO_2_. Fixation and permeabilization were conducted as described above. Cells were rinsed twice with 3% BSA and incubated for 30 min with the Click-iT reaction cocktail, including the Alexa Fluor azide. Cells were rinsed with 3% BSA and DAPI nuclei counterstaining was conducted before imaging. DNA synthesis, or S-phase, was characterized by calculating the fraction of labeled S-phase cells for each condition.

### Proliferation Assay

To determine the effect of altered extracellular environment on proliferation, three treatments were explored: −MMC, +MMC as described above, or +MMC with crowder added 24 hours after seeding. After 24 h, 3 days, and 7 days of culture, cells were fixed, as described above. Cell nuclei were counterstained with DAPI. Nuclei were counted using a 4× objective on an IX-81 inverted fluorescent microscope (Olympus America, Inc.). A minimum of 80 non-overlapping images were obtained for each day of each treatment, ensuring that at least 50% of the total culture area was imaged. The assay was repeated three times for each condition. Cell counts were then normalized to 24 hrs and values are reported as mean ± standard error of measurement.

### Migration (wound healing) Assay

A scratch wound healing assay was performed as described previously [35]. Briefly, MSCs were cultured as described above in a 60 mm-diameter tissue culture polystyrene dish (Falcon 35-1006 Franklin Lakes, NJ) until 70–80% confluence (5–7 days after seeding). Five parallel scratches were created with a P1000 pipette tip. To remove detached cells and proteins after wound creation, cells were rinsed once with fresh media, and new media was added. Dishes were placed in an incubation microscope (IX-81, Olympus America, Inc.) mounted in a WeatherStation live cell environmental chamber (Precision Control Tacoma, WA) and observed over 24 h. Images were obtained using an automated X-Y stage (Prior Scientific Rockland, MA), and analyzed via ImageJ (NIH Maryland, USA) to quantify the number of cells entering the wound site after 0, 14, 18, and 23 h. At least 15 wound sites were analyzed for each treatment and values are reported as mean ± standard error of measurement. To confirm uniform cell densities in each condition, cells were trypsinized after 24 h using 0.25% Trypsin-EDTA (MediaTech Inc., Manassas, VA) for 5 minutes at 37°C and counted via a hemocytometer.

### AFM Imaging and Nanoindentation

Living cells and matrices were imaged and mechanically characterized via atomic force microscopy (AFM; MFP-3D Asylum Research, Santa Barbara, CA) within an inverted optical microscope (IX51, Olympus America, Inc.). Experiments on living cells were conducted in complete MesenCult media ±MMCs at 37°C; experiments on matrices were conducted in PBS at room temperature. Calibration of AFM cantilevers of nominal spring constant *k* = 0.035 nN/nm and probe radius *R* = 25 nm (MLCT, Veeco, Malvern, PA) was conducted as described previously [6]. For each measurement of effective elastic moduli at any given location on any given cell, at least 10 replicate indentations were acquired to maximum depths of 20 nm. At least Five cells were analyzed for each condition. Acquired probe deflection-displacement responses were converted to force-depth responses using measured spring constants and signal calibration factors. Effective Young's elastic moduli were calculated by applying a modified Hertzian model of spherical contact to the loading segment of the force-depth response, as detailed elsewhere [6], with the scientific computing software Igor Pro (Wavemetrics Portland, OR). These values represent the local stiffnesses of the subcellular domains probed in each experiment under contact loading, and are not intended to indicate the elastic properties of the entire cell or the Young's elastic modulus under uniaxial loading. Contact mode imaging of living MSCs and ECM was also performed and orientation analysis was conducted, as described above.

### Statistical Analysis

All data are reported as mean ± standard error of measurement. Unpaired student t-tests were used for comparison for each condition. A value of p<0.05 was considered statistically significant, unless otherwise noted.

## Supporting Information

Figure S1 **Typical distribution of F-actin alignment in mesenchymal stromal cells (MSCs) under induced crowding.** Representative F-actin phalloidin epifluorescent images for human bone marrow-derived MSCs cultured (**A**) −MMC, or without the macromolecular crowder defined in the text; and (**B**) +MMC, after 3 days. Scale bars = 30 µm. (**C**) Representative distributions of F-actin bundle orientation −MMC (blue) and +MMC (red), from which the angular standard deviation was calculated as described in the text. Orientation angle is with respect to an arbitrary axis set; angles −90° and 90° indicate collinear bundles and are thus equivalent. A lower angular standard deviation correlates with a narrower distribution of angles, and is thus indicative of a greater degree of alignment.
(TIF)

Figure S2 **Increase in deposition of fibronectin in presence of macromolecular crowders (MMCs).** (**A**) Western blot of fibronectin secreted by human bone marrow-derived MSCs after 7 days and after 48 hours in culture medium ± MMC demonstrates a significant increase in the cell-deposited fibronectin into the matrix +MMC. (**B**) Densitometry (optical density/mm^2^) of fibronectin bands in **A**. Other crowding agents have been shown to increase the activity coefficient of fibrin, which serves as a crosslinker with fibronectin during clot formation, by an order of magnitude [55].
(TIF)

Figure S3 **Human bone marrow-derived mesenchymal stromal cells (MSCs) uptake of fluorescently labeled Ficoll.** (**A**) TRITC labeled Ficoll (70 kDa, red) and FITC labeled Ficoll (400 kDa, green) remain punctate after endocytosis at 24 hrs and up to 7 days. This finding is consistent with the interpretation that while Ficoll is endocytosed by the cellular membrane, it is not released intracellularly from these vesicles and therefore not capable of providing enhanced intracellular crowding effects. (**B**) Calcein (green) uptake after 24 hrs confirms that Ficoll (70 kDa, red) was not released from vesicles. Scale bars = 20 µm.
(TIF)

Figure S4 **Effects of various macromolecular crowders on FITC-conjugated type-I collagen.** (**A**) FITC-conjugated rat tail type-I collagen (green) deposited on a plasma treated glass bottomed Petri dish at a, 0.01 mg/mL and (**B**) 0.5 mg/mL. This higher concentration is referred to hereafter as FITC-collagen. (**C**) FITC-collagen demonstrates significant alignment in the presence of 62.5 mg/mL of MMCs with a volume occupancy (VO) of 17%, calculated as detailed in [20]. (**D**) Aggregation, but not alignment, of FITC-collagen occurs in solution with 102.8 mg/mL of net negatively charged bovine serum albumin (BSA, Sigma A7906, 17% VO). Adsorption of FITC-collagen is inhibited in presence of (**E**) 0.35 mg/mL of net negatively charged dextran sulfate 500 kDa (DxS, Sigma D8906, 17% VO) and (**F**) 25 mg/mL of DxS (>99% VO). Collagen alignment is observed in presence of (**G**) 4.9 mg/mL of net charge-neutral dextran (670 kDa, Sigma 00896, 17% VO) and (**H**) 25 mg/mL of dextran (87% VO). Note that a qualitative increase in the degree of collagen alignment is observed for 87% VO dextran, which is more comparable to that observed for Ficoll at 17% VO and may be attributed to the corresponding lower concentration of the larger dextran macromolecule. Crosslinking, without effective alignment, occurs in solution with (**I**) 20.5 mg/mL of polyvinyl pyrrolidine (PVP, Sigma 856568, 17% VO) and (**J**) 50 mg/mL of PVP (41% VO). (**K**) Alignment observed in solution of FITC-collagen deposited with 14.3 mg/mL net charge-neutral polyethylene glycol of molecular weight 10 kDa (PEG, Sigma 92897, 17% VO) but not at (**L**) 50 mg/mL (59% VO). Scale bars = 20 µm.
(TIF)

Figure S5 **Expression of the adipogenic marker (PPAR-gamma) is not elevated cultures with macromolecular crowders (MMC).** (**A**) RNA was extracted from monolayers were cultured in basal media for 2, 4 and 7 days in the absence or presence of MMC. Real time PCR was carried out for the adipogenic marker, PPAR-gamma, and we demonstrate no significant difference in expression for control versus crowded cultures. (**B**) Monolayers in basal media after 3 weeks −/+ MMC and stained with Alizarin red. No staining was observed indicating that the crowders do not have intrinsic osteogenic inductive potential. A parallel staining was carried out on monolayers that were chemically induced into the osteogenic lineage. Staining was observed in those wells, confirming that ability of the cells to differentiate and the functionality of the staining protocol (data not shown).
(TIF)
