# Supplementary figures and images for: Macromolecular Crowding Directs Extracellular Matrix Organization and Mesenchymal Stem Cell Behavior
Source: PLoS One. 2012 May 23;7(5):e37904. doi: 10.1371/journal.pone.0037904 (PMC3359376; doi:10.1371/journal.pone.0037904)

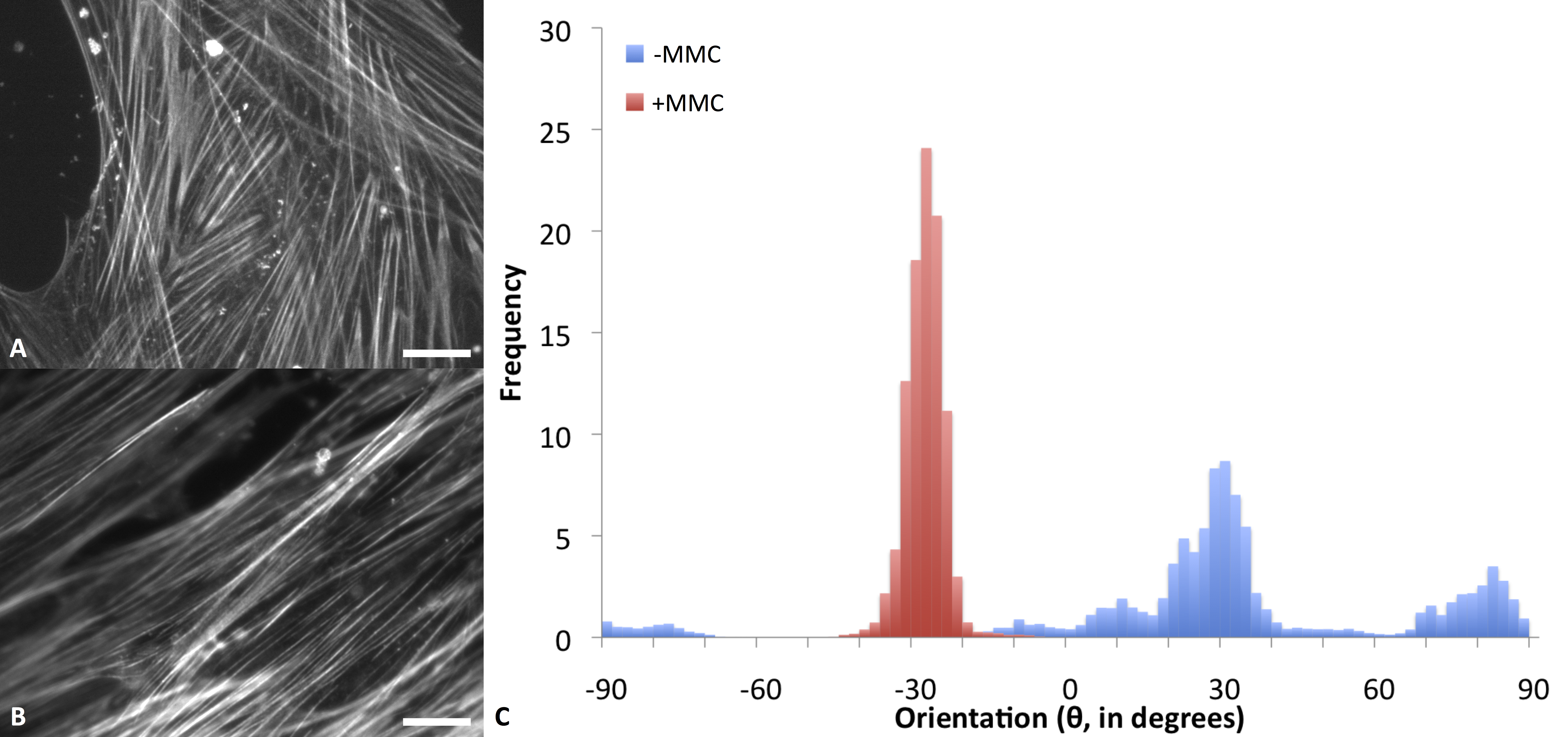

Supplement: Figure S1 — Typical distribution of F-actin alignment in mesenchymal stromal cells (MSCs) under induced crowding. Representative F-actin phalloidin epifluorescent images for human bone marrow-derived MSCs cultured (A) −MMC, or without the macromolecular crowder defined in the text; and (B) +MMC, after 3 days. Scale bars = 30 µm. (C) Representative distributions of F-actin bundle orientation −MMC (blue) and +MMC (red), from which the angular standard deviation was calculated as described in the text. Orientation angle is with respect to an arbitrary axis set; angles −90° and 90° indicate collinear bundles and are thus equivalent. A lower angular standard deviation correlates with a narrower distribution of angles, and is thus indicative of a greater degree of alignment. (TIF) [file pone.0037904.s001.tif]

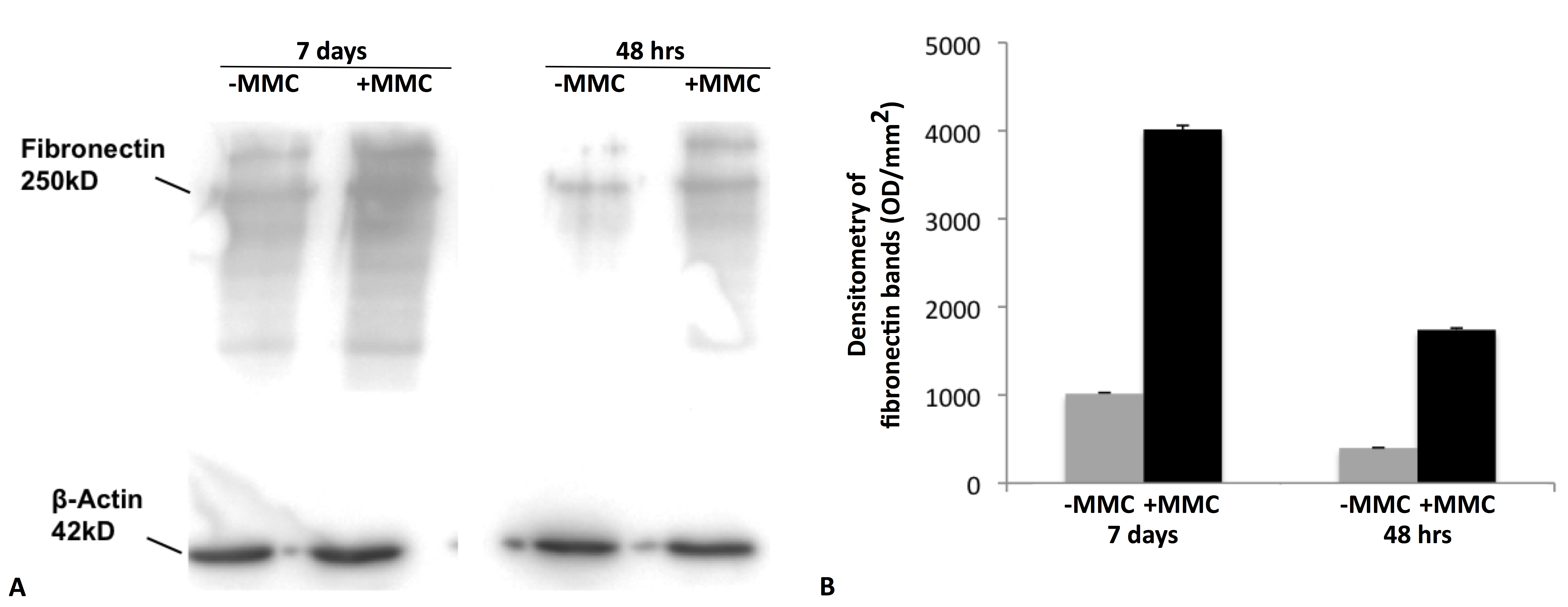

Supplement: Figure S2 — Increase in deposition of fibronectin in presence of macromolecular crowders (MMCs). (A) Western blot of fibronectin secreted by human bone marrow-derived MSCs after 7 days and after 48 hours in culture medium ± MMC demonstrates a significant increase in the cell-deposited fibronectin into the matrix +MMC. (B) Densitometry (optical density/mm2) of fibronectin bands in A. Other crowding agents have been shown to increase the activity coefficient of fibrin, which serves as a crosslinker with fibronectin during clot formation, by an order of magnitude [55]. (TIF) [file pone.0037904.s002.tif]

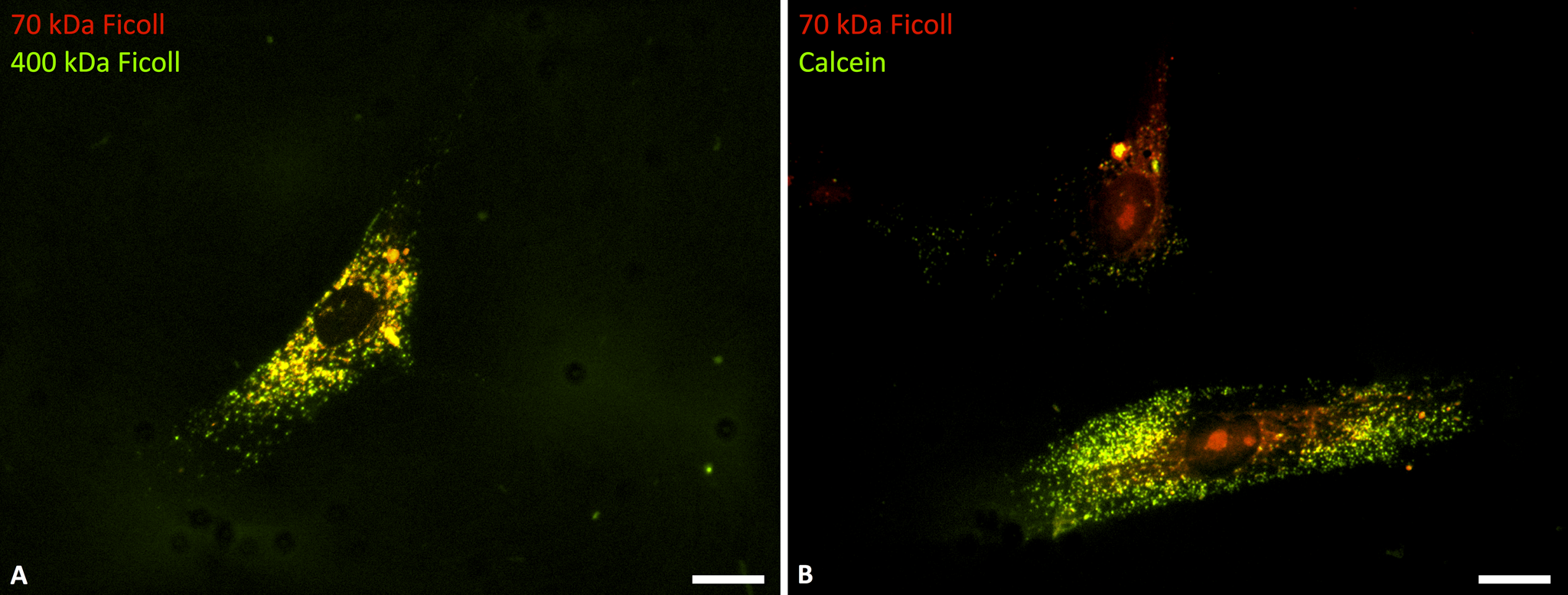

Supplement: Figure S3 — Human bone marrow-derived mesenchymal stromal cells (MSCs) uptake of fluorescently labeled Ficoll. (A) TRITC labeled Ficoll (70 kDa, red) and FITC labeled Ficoll (400 kDa, green) remain punctate after endocytosis at 24 hrs and up to 7 days. This finding is consistent with the interpretation that while Ficoll is endocytosed by the cellular membrane, it is not released intracellularly from these vesicles and therefore not capable of providing enhanced intracellular crowding effects. (B) Calcein (green) uptake after 24 hrs confirms that Ficoll (70 kDa, red) was not released from vesicles. Scale bars = 20 µm. (TIF) [file pone.0037904.s003.tif]

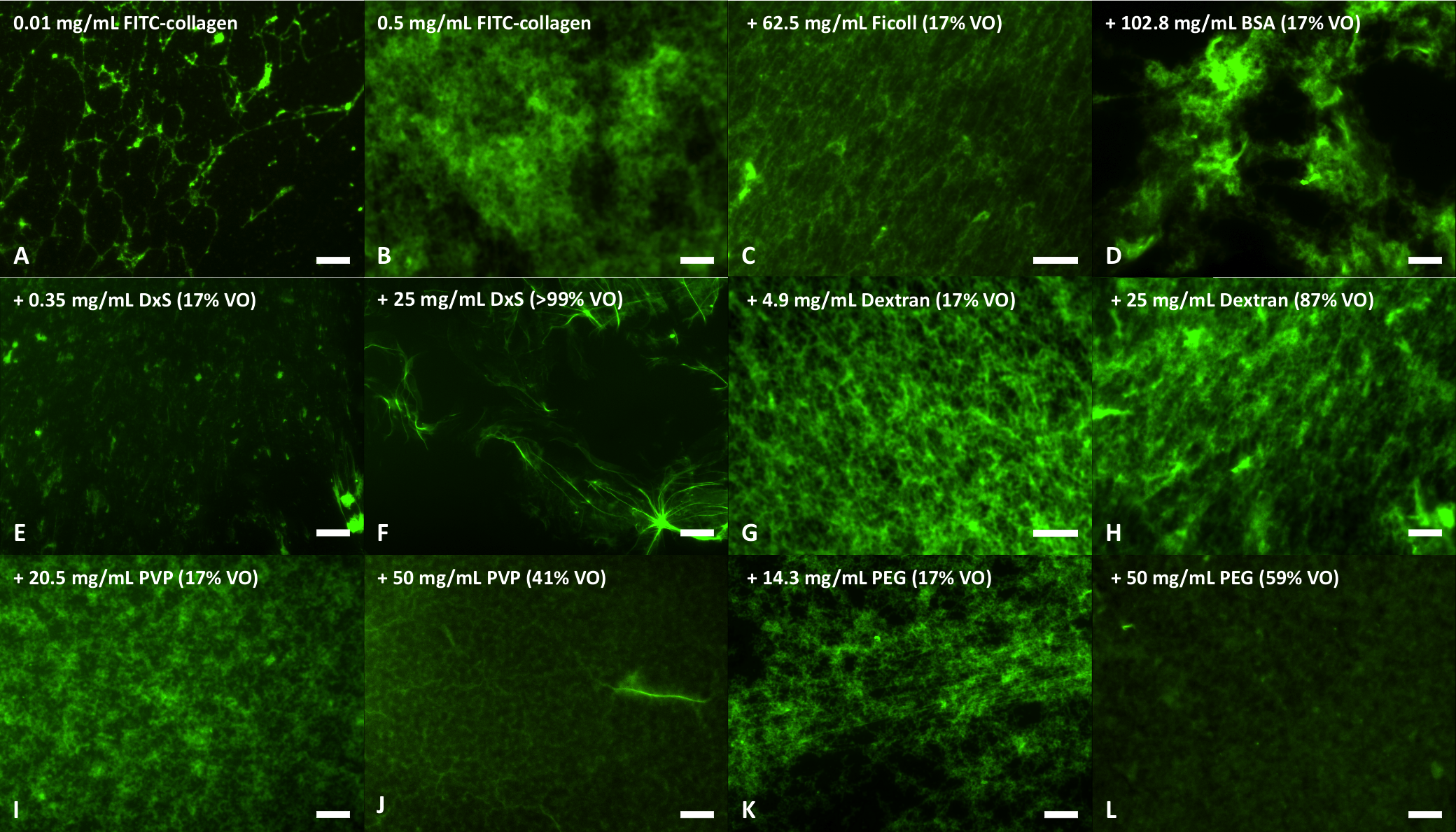

Supplement: Figure S4 — Effects of various macromolecular crowders on FITC-conjugated type-I collagen. (A) FITC-conjugated rat tail type-I collagen (green) deposited on a plasma treated glass bottomed Petri dish at a, 0.01 mg/mL and (B) 0.5 mg/mL. This higher concentration is referred to hereafter as FITC-collagen. (C) FITC-collagen demonstrates significant alignment in the presence of 62.5 mg/mL of MMCs with a volume occupancy (VO) of 17%, calculated as detailed in [20]. (D) Aggregation, but not alignment, of FITC-collagen occurs in solution with 102.8 mg/mL of net negatively charged bovine serum albumin (BSA, Sigma A7906, 17% VO). Adsorption of FITC-collagen is inhibited in presence of (E) 0.35 mg/mL of net negatively charged dextran sulfate 500 kDa (DxS, Sigma D8906, 17% VO) and (F) 25 mg/mL of DxS (>99% VO). Collagen alignment is observed in presence of (G) 4.9 mg/mL of net charge-neutral dextran (670 kDa, Sigma 00896, 17% VO) and (H) 25 mg/mL of dextran (87% VO). Note that a qualitative increase in the degree of collagen alignment is observed for 87% VO dextran, which is more comparable to that observed for Ficoll at 17% VO and may be attributed to the corresponding lower concentration of the larger dextran macromolecule. Crosslinking, without effective alignment, occurs in solution with (I) 20.5 mg/mL of polyvinyl pyrrolidine (PVP, Sigma 856568, 17% VO) and (J) 50 mg/mL of PVP (41% VO). (K) Alignment observed in solution of FITC-collagen deposited with 14.3 mg/mL net charge-neutral polyethylene glycol of molecular weight 10 kDa (PEG, Sigma 92897, 17% VO) but not at (L) 50 mg/mL (59% VO). Scale bars = 20 µm. (TIF) [file pone.0037904.s004.tif]

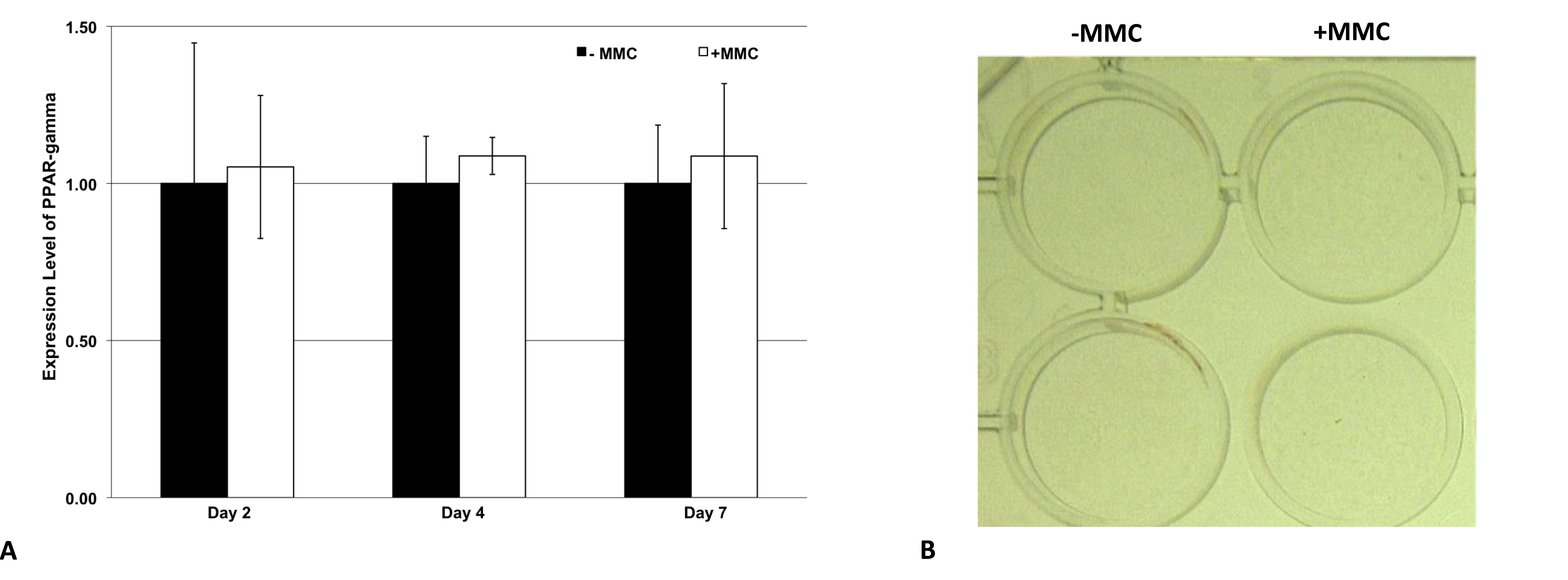

Supplement: Figure S5 — Expression of the adipogenic marker (PPAR-gamma) is not elevated cultures with macromolecular crowders (MMC). (A) RNA was extracted from monolayers were cultured in basal media for 2, 4 and 7 days in the absence or presence of MMC. Real time PCR was carried out for the adipogenic marker, PPAR-gamma, and we demonstrate no significant difference in expression for control versus crowded cultures. (B) Monolayers in basal media after 3 weeks −/+ MMC and stained with Alizarin red. No staining was observed indicating that the crowders do not have intrinsic osteogenic inductive potential. A parallel staining was carried out on monolayers that were chemically induced into the osteogenic lineage. Staining was observed in those wells, confirming that ability of the cells to differentiate and the functionality of the staining protocol (data not shown). (TIF) [file pone.0037904.s005.tif]
